# Some Characteristics of the Tissues Composing and Surrounding the Tooth-root Applicable in the Prevention and Treatment of Infections*Read before the Second District Dental Society, Jan. 9, 1922.

**Published:** 1923-01

**Authors:** Wm. G. Skillen

**Affiliations:** Chicago


### SOME CHARACTERISTICS OF THE TISSUES COMPOSING AND SURROUNDING THE TOOTH-ROOT APPLICABLE IN THE PREVENTION AND TREATMENT OF INFECTIONS*

BY WM. G. SKILLEN, D. D. S., CHICAGO

The impossibility of going into a full description of the tooth and its investing structures where time is limited, is obvious. The essayist has, therefore, selected such of the characteristics of these structures as seem to be of greatest importance or interest. It should go without saying, that our ultimate aim is further than this—indeed, not to stop until we have as complete an understanding of these structures as is mortally possible.

Wherever feasible, the features brought out here are correlated with the pathological or the clinical side of the subject.

---

* Read before the Second District Dental Society, Jan. 9, 1922.

The ideas presented here, are, in a brief way, the sum total of our knowledge today. These ideas are available from the writings of the past or present, so that, for the sake of conciseness, references, with the exception of one or two, have been omitted.

### THE GINGIVUS

The gingivus is that part of the gum tissue which extends above the crest of the alveolar process and surrounds the gingival portion of the tooth. It is composed of that type of epithelium which covers the surface of the body and lines the mouth cavity—namely, stratified squamous—a name which implies that this epithelium is made up of several layers of cells with one or more of the surface layers flattened or scale-like. The epithelium rests upon a papillated stroma of fibrous tissue. The papilli of connective tissue contain blood vessels and lymphatics that furnish nutrition and drainage for the epithelium into which they extend more or less deeply. Stratified squamous epithelium is particularly well suited for the role of protection, in that it responds quickly to stimuli, increasing in thickness and density, or, by production of new cells replaces quickly those lost by abrasion, so that the underlying tissues may be covered by a protective, sealing membrane.

On the outer surface of the gingivus, the protective qualities of the epithelium are enhanced by a hardening—a keritanizing, of the surface layers, forming thus a *stratum corneum,* a structure especially characteristic of the palms of the hands and the soles of the feet. This hardened layer is found, as has been said, on the outer surface of the gingivus and usually ends at the tip of the free gingivus, or, more commonly, the free margin of the gum.

The continuation of the above epithelium—stratified squamous—to line the gingival space or crevice, does not, therefore, exhibit such a protective arrangement, indicating that this area is not so resistant to abrasion and infection, as the outer surface. The difference in the structure of the epithelium on the outer and inner surfaces of the free margin is of course due to the naturally secluded position of the latter—the inner surface. The effects of this seclusion are further exemplified by the thinness of the epithelium in the gingival space as compared to that on the outer surface; and by the largeness and the looser arrangement of the cells composing the epithelium. The papilli, too, at times extend very deeply into the epithelium, so that their tips are covered frequently by only one or two cells, and occasionally, none at all, so that breaks, as pointed out by Dr. Box, occur in the protective epithelial covering.

At the bottom of the crevice, only the close adaptation of the epithelium to the cementum and dentin protects the underly tissues. It should be understood that at no time is the epithelium fused with the adjacent hard tissues; it simply lies in close contact with them. Therefore, there is always a break, although usually very minute at this point, in the general continuity of the epithelium which is only minimized by the snug fit of the free margin of the gum around the neck of the tooth. This adaptation of the tissue to the tooth surface, is maintained by, and depends upon, the normal tension of the first, the free gingival group, of peridental membrane fibres. This group of fibres is inserted in the cementum as the cervical margin, runs occlusally and outward to the anastomosis with the fibrous tissue of the gingivus.

Practical considerations are obvious. Every means should be exercised to maintain the natural snug adaptation of the free margin of the gingivus to the neck of the tooth. The greatest diligence should be used in detecting or in preventing distortion of this tissue and emphasis should be placed on the latter procedure.

### THE ALVEOLAR PROCESS

The alveolar process may be defined as that portion of the bone of the jaw which surrounds the root of the tooth, but, on the average, is short of the cervical line by about 2 mm. Its growth is an accompaniment of tooth development. The process has the function of affording attachment for the bulk of the fibres constituting the peridental membrane.

The subservience of the process to the tooth is an important consideration. Changes in the position of the latter will lead to changes in the former. Removal of the tooth will result in the absorption of the process and destruction of any part of the peridental membrane is followed by removal of that part of the bone with which those fibres are continuous. In the words of Dr. G. V. Black, the alveoli are made to fit the teeth and the process forms the alveoli. It is clear, therefore, that restoration of the process depends upon the return to function of the peridental membrane.

The structure of this bone with the relationship described bringing about constant change, therefore, defies description. At times it presents a more or less medullated, i. e., spongy or cancellated appearance, and again takes on a more compact character. Like all bone it is subject to physiological absorption and repair. It has a rich blood supply, a rich collateral circulation, many vessels passing through the medullary spaces or Haversian canals to anastomose with those coursing through the peridental membrane and the gum tissue.

### THE PERIDENTAL MEMBRANE

Excluding the first or free gingival group of fibres, the remaining solid mass of fibres of the peridental membrane attached to the cementum at one end, pass outward to be inserted into the approximating tooth or the bone of the alveolar process. The greater mass is attached to the process. These fibres are so arranged about the whole root of the tooth, as to resist stress from all directions. This arrangement is one of such beautiful adaptation that if considered with the other functions of the membrane, namely—upkeep of cementum and alveolar process, it at once hints at specialization. To prevent destruction to the smallest degree of a structure bearing this attribute, is a thing to be learned and appreciated by all.

Coursing through the membrane, about midway between the cementum and the alveolar process, are groups of blood vessels and nerves surrounded by a small amount of fibrous tissue—the so-called interstitial tissue. For the most part, the vessels run parallel to the long axis of the tooth. To the outerwalls of these vessels and nerve groups, are attached the minute lymphatic vessels which drain the gingivae and peridental membrane.

The general principals upon which the lymphatics function should be remembered. Serum is delivered by the minute capillaries of the blood system to all the tissues of the body to serve as their nutritive fluid. This fluid or lymph seeps through all the interstices of the tissues and after being added to by the products of metabolism of the cells, is drawn into lymph spaces and channels and returned to the blood stream. In conjunction with the blood stream, the lymphatics afford drainage for the tissues. As the lymphatics accompany the blood vessels, we must note the distribution of the latter.

The blood supply of the peridental membrane is threefold. Vessels pass through the gum tissue on the outer surface of the alveolar process, arch over the crest of the process and pass down through the membrane toward the apex. Others, the main supply, enter the membrane about the apex and pass through the membrane toward the gingivus. The third group reaches the membrane through the alveolar process, a rich anastomosis existing between it and the membrane.

The lymphatic channels accompany these vessels, the flow of the lymph being from gingivus to apex. Those lymphatics which have their beginning under the epithelium lining the gingival space and which drain the tissues about the neck of the tooth, pass through the membrane toward the apex, generally parallel with the long axis of the tooth. Those which drain the outer surface of the gingivus pass through the gum tissue on the outer surface of the alveolar process.

The course of the vessels through the membrane appears to bear a relation to the extension of infection into the investing tissue. Pocket formation as pointed out by Dr. Black may be and frequently is, unilateral or of unequal depth. Furthermore, infections or irritations which affect the outer surfaces of the gingivae, such as salivary calculus, do not cause formation of pockets in the peridental membrane, whereas those which involve the inner surface, for instance, serumal calculus may or do cause destruction of the membrane. Hatton has also pointed out that inflammatory reaction progresses along the vessels and Noyes that the first changes occur in the same locality.

### CONTACT POINT OF PULP AND PERICEMENTUM

At the apex, the fibrous tissue of the peridental membrane which surrounds and is imbedded in the root, extends for a short distance into the foramen or foramina, of the tooth root where it becomes continuous with the pulp of the tooth. A gradual change in the character of the tissue takes place, the fibrous type characteristic of the membrane giving way to the embryonic type peculiar to the pulp. At just what point the transition takes place has not been determined; probably when the canal is large the change occurs at about the dento-cemental junction. The difference between these two tissues should be remembered. The fibrous tissue which composes the peridental membrane is dense and strong, rope-like in character. The tissue composing the pulp is cellular, and soft; jelly-like in consistency. The removal of the latter (the pulp) is not fraught with difficulty as the former whose natural resistance of tearing may be augmented by attachment to the cementum in its course through the foramen. Hence, it is very likely that most of this tissue remains behind in the foramina when a pulp is extirpated.

### CLOSURE OF ROOT FORAMINA

At this point it might be well to consider the subject of root foramina obliteration, a subject which has given rise lately, to some furor of interest. It is clearly obvious that any reaction on the part of the tissue surrounding or filling the foramina depends upon the maintenance of its vitality. Given a vitality it would be not at all strange that one or more of the foramina should be partially or completely obliterated, especially in the presence of a stimulation such as a low grade inflammation would set up. At the present time, however, there is not sufficient evidence to indicate that partial obliteration is a general sequence of pulp infection or pulp removal, let alone a complete obliteration. We should bear in mind that what may cause the formation of calcifications, instead, frequently cause absorptions. Calcifications within the pulp canal where pulp tissue rules may, as Salter pointed out back in 1873, exhibit many variations and alterations in structure—formations of a dental, or a cemental, or a homogeneous character appearing. Where peridental tissue fills the foramina, we should expect to find deposits of a cemental type although these too may assume a structureless form. The essayist is of the opinion that most of the secondary deposits found in pulp canals occur before pulp removal rather than after, and therefore, are not to be depended upon for sealing root ends. If, however, any hopes are to be realized along this direction radical methods of treatment must cease, if there is not already sufficient ground for their discardence.

### CEMENTUM AND DENTIN

The cementum is a calcified tissue which, commencing at the gingival line, surrounds the dentin of the root of the tooth. Its function is to afford attachment for the peridental membrane around whose fibres cementation takes place. The fibres become more or less calcified along with the matrix, and are termed usually "the embedded fibres."

Cementum varies considerably in thickness and structure, scarcely two cases being alike. In general, however, this tissue on the gingival third of the root appears structureless unless uncalcified embedded fibres appear, and is extremely thin. Its thickness has been well compared by Dr. Box, to a hair of the scalp. From this point to the apex, the cementum becomes progressively thicker and cellular. Usually the thicker the tissue becomes, the more cells will be seen. The cells, cement corpuscles or embedded cementablasts, occupy spaces, lacunae in the calcified matrix. Cytoplasmic processes of inconceivable variety of form ramify throughout the matrix and anastomose more or less completely with each other, by means of camaliculi. In this particular only does cementum in any way resemble bone. In the irregularity of number, size and form of the cells and their processes; in the irregularity of arrangement of cells and lamellae; and particularly in the absence of nutrient canals, there is no resemblance whatever between cementum and bone.

In the absence of blood vessels, cementum, in the words of Dr. G. V. Black, has not in itself the power of initiating or carrying forward any reparatory process. Absorption and repair depends wholly upon the peridental membrane, as does chiefly, its nutrition.

Cementum appears to be an extremely porous tissue. This characteristic seems to be due, not altogether to the presence of cells or embedded fibres, but to the fact that its matrix frequently appears to be very poorly, or incompletely, calcified. This feature does not always show in ordinary preparations. If, however, the tooth in toto is stained so that the results of the coloration must be due to the stain having infiltrated through the tissue, these areas, when present, as well as all the usual elements of cementum are revealed. Due to this ability on the part of the stain to penetrate the substance of the cementum throughout its extent, this tissue should be considered as being very permeable and so subject to infection.

Staining by this method, furthermore, seems to show that cementum and dentin are not separated from each other by an impervious layer of tissue usually described as existing in the gingival region. A study of developing and adult teeth seems to indicate that the normal arrangement is for the dentinal-tubules to extend to the dento-cemental junction. Subsequently, there may be a calcification of the tubules at the junction over a part of the root, but this in many cases seems to be only partial, the fineness, then, of the tubules rendering them indistinct in the ordinary preparations.

If, therefore, the cementum is a porous tissue and abuts upon the tubules of the dentin, or the granular layer of the dentin with which the tubules are continuous, there seems to be nothing to prevent fluids or infection from passing from one to another. In this connection, it is interesting to note that most tubules and their finer branches may be viewed at 100 diameters, while it requires 1,000 diameters to successfully see most bacteria.

### DENTIN AND CEMENTUM AT THE APEX

In the apical region the continuity of dentin and cementum is very seldom disputed. The intermingling of the structural elements of the two tissues seems to be a consistent feature. Very frequently this arrangement is further augmented by absorptions dipping deeply into the dentin, the lost tissue being replaced by cellular cementum.

This conception of the porosity and continuity of dentin and cementum over the whole surface of the root, is substantiated somewhat by an experiment in which both tissues have been stained by permitting the stain to pass only through the cementum. Regardless of the thickness of the cementum, the stain has passed through that tissue and into the dentin. In the apical region where the wall of dentin is thin due to its tapering form, the stain applied in the above manner has usually reached the canal. At the dento-cemental junction, all the tubules with their myriad of branches have been impregnated and a somewhat diffused coloration of the dentin matrix has occurred. These results have been quite different from those obtained by Marshall who attempted the same thing via the pulp canal but who failed to obtain a passage of the stain beyond the dento-cemental junction. More complete evidence must be sought on this subject.

However, considering the thinness and apparent porosity of the cementum in the gingival area and the relation of the tubules to the cementum, the essayist is of the opinion that the dentin very likely becomes involved by the diffusion of infection through the cementum. The extent of involvment being uncertain, and probably varying, the efficacy of scaling or polishing procedures would seem to be very uncertain.

In the apical region suppurative involvment of the investing tissue, or the tissue contents of the foramina, would seem to be sure to involve more or less of the cementum, and probably also the dentin. Particularly, too, in this region where the wall of dentin is thin, especially evident in molars, and where so often there apparently exists a perfect anastomosis of the elements of dentin and cementum, would suppuration of the cytoplasmic contents of the dentinal tubules be likely to involve more or less of the cementum.

For the same reasons, we should not overlook the effects of medicinal or chemical treatment.

### CONCLUSIONS

In conclusion, the essayist wishes to point out, that much has yet to be done in the way of correlating the findings of the three departments of endeavor—the anatomical, the pathological and the clinical. Indeed, much has to be done in each of these alone.

And until every fact is correlated, until it can be said with certainty that such are important, or so and so unimportant, every fact concerning the tissues involved should be given full consideration. The fundamentals, tissue structure and physiology, should be understood to the fullest. Without this knowledge it is impossible to interpret other findings. What if these findings appear to make the task impossible? The one sure method of treatment available, namely "Prevention," must be exercised.—*Dental Items of Interest.*

### DISCUSSION OF DR. SKILLEN'S PAPER

*President Gough*—We regret exceedingly that Dr. Black at the last moment could not be with us. We fully expected him to be here to give us the practical application of Dr. Skillen's paper. Even when Dr. Skillen left Chicago on Saturday he thought Dr. Black was coming, but I received a message from Dr. Black this morning, saying it would be impossible for him to attend the meeting, and that he wanted to explain fully to the members of the Second District that this was one of the very few occasions when his name had appeared on a program and he had to be absent.

We are fortunate in having on our own list of members, Dr. Victor Stoll, who will discuss this paper of Dr. Skillen.

*Dr. Victor Stoll*—Mr. President, Ladies and Gentlemen: I have rather a difficult job on hand. I am somewhat confused. I did not have the slightest idea of the presentation of Dr. Skillen, but I took a chance. In looking over the literature I have found in the *National Dental Journal* of last year that Dr. Skillen spoke before the Dental Society of Chicago. It was a symposium, in which Dr. Skillen took part. I acquainted myself with the ideas in this article, and then I acquainted myself with some of the ideas of Dr. Black, and I thought I was ready to take part in the discussion of tonight; but I find myself in rather a peculiar position, as tonight's presentation varies from the ideas in the article. I am not an histologist. I am a mere dentist, a clinician, and I have an interest in histology and all laboratory work only from the clinician's standpoint: "How much does it help me in my daily work? How much does it explain my failures and my successes?"

I want to make certain that I understood Dr. Skillen. You substantiate the opinions of Dr. Boedecker, Dr. Box, Dr. Heitzmann and many other histologists of the old school who believe the make-up of the tooth is such that the different structures of the tooth inter-communicate with each other. I must confess that the ideas expressed tonight, presented as the findings in the laboratory, do not suit my fancy and my work as a root canal worker. I would rather picture, according to the results I get, some barrier between the dentin and the cementum. I could almost imagine it, and, strange to say, after making a study of the latest research, by such men as Dr. Noyes, Dr. Marshall and Dr. Hopewell-Smith, of international reputation, I found my imagination almost true. They definitely state that they have never succeeded in passing this barrier, this homogeneous, structureless mass between the cementum and Tome's granular layer, using the most ingenious methods of staining. Dr. Marshall, after carrying out a number of careful experiments of staining normal teeth in vacuo and in vivo on albino mice, comes to the conclusion that those who have succeeded in getting a stain through either made errors in technique or did not use a normal specimen. Practically these views work out very well for me—clinically. Why? In conservative treatment of pulpless teeth we are greatly concerned with the eradication of infection and making the tooth safe for general health. What is generally the course of infection in a tooth? It travels through the canal and settles at the apex, but very seldom on the sides of the root, and when it does settle at the sides it mostly comes from the gingiva along the peridental membrane, but not from the canal. Therefore, it gives me the idea that infection and its products do not penetrate through the sides of the root, but travel along the root canal towards the apex. Therefore, when we close up the canal we also block up the main road for infection towards the interior of the body, and we find our results of root canal operations highly satisfactory.

Another instance of clinical experience: When we put into the tooth such a violent drug as arsenic, if there were an inter-communication through and through, we would have a necrosed socket very soon. Dr. Preiswerk, a German authority, who made a study of what happens with this violent drug, has found it to permeate the dentin, but is stopped at the dento-cemental junction.

The question is, how can we correlate these facts of our practice with the findings of the laboratory?

I can frankly and openly state that we do get wonderful results in all kinds of cases, even considered by many as hopeless. My greatest surprises I have received when I have attempted to treat pulpless teeth in sick individuals, where everyone thinks it should not be done. I wanted to convince myself about the results in such cases, and if we accomplished nothing we still could use the forceps. I assure you that there have been some remarkable cases which taught me a lesson not to sacrifice teeth so readily. If I may cite to you one of many cases (the patient lives to tell the tale and is ready to do so): A lady came to me who had suffered with gallstones for five or six years, with the typical appearance of jaundice. There was no question of it in the diagnosis. She came to me after she had made an appointment with the celebrated surgeon Dr. Berg to be operated on. In the meantime she had radiographs taken for her internal trouble, and the radiographer advised her to have radiographs made of her jaws. He found a great deal of infection and told her so. She came to me for advice. She asked if she should first attend to her teeth and then be operated on, or first be operated on and then have her teeth attended to. I never suspected what the outcome would be, but I said: "On general principles you would be wise to have your teeth attended to first. If you will have your teeth and your masticatory apparatus put in good shape it will help to restore your health." She took my advice. I extracted a few broken down teeth, and I treated a number of pulpless teeth, and the result was remarkable. Three years have passed since and the change in her health I cannot describe to you. Dr. Berg is still waiting to perform the operation.

If there were a thorough inter-communication between the structures of the tooth it would be impossible for me to obtain such remarkable result because the sealing of the canal would not prevent the infection from the dentin from penetrating into the surrounding tissues. In this case apparently the dental infection was a contributing factor for the systemic condition. The case cleared up under conservative methods and I am sure that Nature herself with her many protective measures invites us to do root canal work.

If you will permit me I will read to you a little discussion I had prepared, taking for granted that Dr. Black would be here and had shown you the application of tonight's work to root canal work.

It was disheartening to see to what pitiful state the hysteria of focal infection has brought dentistry. Dentistry was bankrupt. The valuable organs of mastication were slaughtered in the name of an unfounded theory and we were going backwards to the time when pulling a tooth was the only remedy for all dental ills. Why then the study of so many sciences for so many years, if all modern, elaborate methods of diagnosis wind up in extracting the teeth and crippling the masticatory apparatus beyond recognition and then replacing the losses with substitutes that are far from efficient? But it seems to me that a revival is taking place and we are coming back to dentistry that ought to occupy a prominent place in the healing art; to dentistry that heals and conserves.

The papers reported: "In Chicago some one turned in a false fire alarm, and the result was a few deaths and many injured."

I cannot warn you enough against the false infection alarm about pulpless teeth that has crippled many a mouth unnecessarily and caused a great deal of confusion in the ranks of our profession.

What would you think of one who turns in a fire alarm when he sees a candle or a gas light burning? He sees fire burning, and the elements are very dangerous and destructive. So is infection. That local infection in any part of the body may be of serious consequence to general health is so fundamental that it needs no discussion. Wherever infection is found it should be eradicated.

Scientifically the study of infection is a very intricate one and keeps busy the greatest minds. It is the most difficult problem on account of the biological factor, that no one can reproduce in the laboratory. The rabbits, the dogs, the guinea pigs, though animals, are by no means human beings, with the same biological qualities, and the artificial culture media in the test tubes are very far from the normal growth and qualities of the micro-organisms in the human body. Very often, therefore, the laboratory deductions do not coincide with clinical results. It is true that the laboratory researches have contributed a great deal to our knowledge in all fields of science. But to give the laboratory the leadership in decisions of practice, sweeping aside all clinical experience, is very dangerous. To me it appears that the laboratory must aid the clinician in explaining the phenomena he sees in his practice, his failures as well as his successes. A theory, based on laboratory findings only, that does not help the clinician in all phases of his work, no matter how beautiful philosophically, is of no value in practice.

From a laboratory standpoint a pulpless tooth contains infection and is therefore a menace to health.

At a certain meeting a gentleman stated that 98 per cent. of pulpless teeth are infected and should therefore be extracted. I also heard Dr. Rosenow state that all pulpless teeth are infected and he would rather see the people gum their food than chew it with pulpless teeth.

This is the stand of the extreme focal infection advocates, based on laboratory findings. Is it clinically true? No. It is a half truth and very misleading. The finding of micro-organisms, even virulent, in a tissue does not mean infection at all. You can at all times obtain cultures of a majority of micro-organisms from our bodies. You can also obtain cultures from everything we eat and drink and still we are not considered to be infected. Sir Kenneth Goadby found pure cultures of micro-organisms in amputations of seven or eight years standing and the general health of these individuals was not affected in any way.

It is only under certain conditions of the body that certain micro-organisms with certain qualifications produce symptoms of a disease we call infection. Infection is a definite bio-chemical reaction of the body cells to a toxin (foreign protein), the products of micro-organisms. It may manifest itself in three ways—first, sensitizing; second, exciting, and third, vaccinating.

Normally the body is well equipped with a number of wonderful mechanisms to destroy the infection and bring about immunity. In this case the infection has a vaccinating effect. Vaccination is the biological method of treating infection, and it is based on the stimulation of the normal defensive forces of the body to destroy the infection. And this is my view on root canal operations. I look upon root canal treatment as a form of auto-vaccination for the purpose of auto-immunization, and it is in accord with all the present day knowledge of the subject. A root canal operation properly carried out brings about an inverted resistance of the apical tissue to the infection present and helps in the elimination of the infection by the stimulated normal body forces and the protective histological arrangement of the dental tissues. The idea of removing all dental foci of infection surgically before attempting to eliminate them medicinally is, in my mind, wrong.

I assure you that there is very little danger in a pulpless tooth properly taken care of. Clinically I am convinced that conservative treatment of pulpless teeth is beneficial to the general health, and scientifically evidence begins to come in from many reliable sources in a very positive and confirmative way.

The latest researches of many prominent histologists show us that the arrangement of the structures of the tooth is protective in nature against the spreading of infection.

Within the study of immunity lies the solution of the problem about the pulpless tooth.

The positive knowledge of all the forces that counteract infection, be it by structure or by bio-chemical means, will remove the scare from the profession about the pulpless tooth.

*Dr. Ottolengui*—Why does the result of your treatment clinically make you sad when you hear that there is communication between the cementum and the dentin? What is it that you do in a tooth that you could not do just as well if there were inter-communication?

*Dr. Stoll*—We deal with infection and its products that are soluble poisons. If we assume the inter-communication of the dentinal tubules through the cementum with the surrounding tissues as a fact, the operation of filling root canals becomes a very dangerous procedure, and I would be the first one to discontinue the practice of it. The dentin, with its labyrinth of dentinal tubules, once infected, would always be an active focus of infection nourished from the inside. Even if we could disinfect the dentin it would be temporary, as the filling of the root canal would only block the ingress of infection from the oral cavity, but would leave the dentin open to reinfection through the circulatory system. If there is inter-communication there is constantly a source of infection that I may not be able to remove, and it therefore comforts me more to see the infection surrounded by an impenetrable barrier that will exhaust the infection in time for lack of food or make it latent. If I have a barrier from the inside and I block up the canal through which the infection may travel from the outside I am safe, and the satisfactory results clinically prove it.

I thought Dr. Skillen would bring something to substantiate my views; therefore had started my written discussion with the words, "How pleasant it is to hear, etc." (Laughter.)

That does not mean I am not happy to hear the truth. But the argument about the inter-communication of the dental structures is not a new one and is far from being considered settled by the demonstration of staining a tooth through and through in the laboratory. It seems to me that recent researches confirm the view that the dental tissues are so arranged that infection cannot travel through.

*Dr. Skillen* (in closing)—There is a question that is very doubtful—whether a lot of things that we have got to solve will ever be solved. The question arises, which will be the most important to consider, the easiest way or the most difficult? I think it will be the most difficult proposition to be considered, to be on the safe side and to be honest with yourself.

I want to correct Dr. Stoll on one point—the arrangement of the fibres around the periapical end has nothing to do with the direction in which infection spreads. Infection spreads along the blood vessels or lymphatics. They spread in a fan form and penetrate the bone, and that is why you do not have the infection in other directions. The lymphatics drain the pulp tissue and the peridental tissue from the apex.

When a certain portion of the apical socket is gone from the jaw the filling-in processes do not indicate that there is a re-attachment or that your infection is forevermore done away with. Again, you do have perfect root canal fillings, and maybe six months or a year or two later, your tooth is lost, and you cannot tell why. What does it? I do not know.

The society passed a vote of thanks to the essayist. And then adjourned.—*Dental Items of Interest.*

